# Performance Changes Following Heat Acclimation and the Factors That Influence These Changes: Meta-Analysis and Meta-Regression

**DOI:** 10.3389/fphys.2019.01448

**Published:** 2019-11-27

**Authors:** Courteney Leigh Benjamin, Yasuki Sekiguchi, Lauren Amanda Fry, Douglas James Casa

**Affiliations:** Department of Kinesiology, Korey Stringer Institute, University of Connecticut, Storrs, CT, United States

**Keywords:** training, adaptation, thermoregulation, athlete, capacity

## Abstract

Heat acclimation (HA) is the process of intentional and consistent exercise in the heat that results in positive physiological adaptations, which can improve exercise performance both in the heat and thermoneutral conditions. Previous research has indicated the many performance benefits of HA, however, a meta-analysis examining the magnitude of different types of performance improvement is absent. Additionally, there are several methodological discrepancies in the literature that could lead to increased variability in performance improvement following HA and no previous study has examined the impact of moderators on performance improvement following HA. Therefore, the aim of this study was two-fold; (1) to perform a meta-analysis to examine the magnitude of changes in performance following HA in maximal oxygen consumption (VO_2max_), time to exhaustion, time trial, mean power, and peak power tests; (2) to determine the impact of moderators on results of these performance tests. Thirty-five studies met the inclusion/exclusion criteria with 23 studies that assessed VO_2max_ (*n* = 204), 24 studies that assessed time to exhaustion (*n* = 232), 10 studies that performed time trials (*n* = 101), 7 studies that assessed mean power (*n* = 67), and 10 papers that assessed peak power (*n* = 88). Data are reported as Hedge's g effect size (ES), and 95% confidence intervals (95% CI). Statistical significance was set to *p* < 0.05, a priori. The magnitude of change following HA was analyzed, with time to exhaustion demonstrating the largest performance enhancement (ES [95% CI], 0.86 [0.71, 1.01]), followed by time trial (0.49 [0.26, 0.71]), mean power (0.37 [0.05, 0.68]), VO_2max_ (0.30 [0.07, 0.53]), and peak power (0.29 [0.09, 0.48]) (*p* < 0.05). When all of the covariates were analyzed as individual models, induction method, fitness level, heat index in time to exhaustion (coefficient [95% CI]; induction method, −0.69 [−1.01, −0.37], *p* < 0.001; fitness level, 0.04 [0.02, 0.06], *p* < 0.001; heat index, 0.04 [0.02, 0.07], *p* < 0.0001) and induction length in mean power (coefficient [95% CI]; induction length 0.15 [0.05, 0.25], *p* = 0.002) significantly impacted the magnitude of change. Sport scientists and researchers can use the findings from this meta-analysis to customize HA induction. For time to exhaustion improvements, HA implementation should focus on induction method and baseline fitness, while the training and recovery balance could lead to optimal time trial performance.

## Introduction

Athletes in team and individual sports use a variety of training methods to achieve peak performance. One training modality that has been established is known as heat acclimation (HA) (i.e., training in a hot, artificial environment) or heat acclimatization (i.e., training in a hot, natural environment), repeatedly. HA is the process of intentional and consistent exercise in the heat that results in several positive physiological and perceptual adaptations (Armstrong and Maresh, 1991). A decrease in heart rate, internal body temperature, sweat electrolyte concentration, and perceptual measures and an increase in sweat rate and plasma volume are all positive adaptations that occur throughout HA (Périard et al., 2015; Casadio et al., 2017). While the physiological benefits of HA have been established for many years (Adolph, 1938), a growing body of literature has emerged investigating the many performance benefits of HA in hot and thermoneutral environmental conditions. While the physiological and perceptual benefits of HA are the mechanisms behind enhanced exercise performance, actual result from competition, such as a faster race time, an increased time to exhaustion, or improved aerobic capacity are typically the primary outcomes.

Even still, understanding the mechanisms behind enhanced exercise performance is critical to adopting optimal training programs. Cardiovascular adaptations, including decreases in heart rate and increases in plasma volume, occur within 3–6 days of HA and are known to have a strong influence on exercise performance (Sawka et al., 2011; Périard et al., 2016). Body temperature adaptations, both internal and skin, also occur within 8 days of HA (Armstrong and Maresh, 1991) and are known to improve exercise performance (Nybo and González-Alonso, 2015). Decreases in sweat electrolyte concentration typically occurs within 5–10 days of HA and increase in sweat rate typically occurs within 5–14 days of HA (Armstrong and Maresh, 1991). These adaptations can enhance exercise performance in a hot environment, as these mechanisms improve thermoregulation (Nuccio et al., 2017). These adaptations independently and collectively improve exercise performance by helping the body thermoregulate more efficiently and reduce the overall physiological strain.

In the literature investigating HA, “performance” has been used to describe both physiological and perceptual improvements within a relative bout of exercise. “Performance” has also been used to describe the outcomes from direct measurements, such as time trial and maximal oxygen consumption (VO_2max_). For this meta-analysis, performance will be defined as the result of any established test that measures exercise ability. Common tests that have been used to assess exercise performance following HA include: VO_2max_, time to exhaustion, time trial, mean power, and peak power.

While the many physiological and performance benefits of HA have been reported in the literature, there are several methodological discrepancies in the literature that could lead to increased variability in these results, including fitness level, induction length, session duration, exercise intensity, induction method, induction length, environmental conditions of induction, and environmental conditions of testing. Induction method and exercise intensity typically refers to isothermal, controlled work-rate, or self-paced exercise (Daanen et al., 2018). The isothermal induction method involves having individuals exercise to achieve a critical internal body temperature threshold (typically 38.5°C) and maintain that temperature or higher for at least 1 h by adjusting the exercise intensity (Taylor and Cotter, 2006; Taylor, 2014; Périard et al., 2015). The controlled work-rate method involves individuals exercising for a constant intensity for a set duration (Tyler et al., 2016). Both of these methods result in various physiological responses and are thought to influence HA results (Périard et al., 2015). HA induction length refers to the number of days an individual is exposed to exercise in the heat. Previous literature defined various HA protocols as short-term (<7 days), medium-term (8–14 days), and long-term (≥14 days) and concluded that some physiological adaptations (internal body temperature and heart rate) can occur from a short-term HA protocol, however, the extent to which these adaptations translate to specific performance tests are unknown (Tyler et al., 2016). Previous investigations sought to gain a better understanding of induction method and length and reported that isothermal and controlled work-rate protocols yielded similar adaptations and that length did not contribute to additional adaptations (Gibson et al., 2015a,b). Session duration refers to the time per each HA session and typically ranges from 60 to 120 min, with previous research favoring increased duration for physiological benefits (Sawka et al., 2011). In addition to these considerations surrounding HA induction protocols, individuals with high fitness are generally more tolerant to heat and this factor could play a role in the magnitude of performance enhancement from HA (Pandolf et al., 1977; Gardner et al., 1996). Furthermore, environmental conditions during HA induction and testing can modify the response to exercise in the heat and change the magnitude of adaptations to HA, most likely due to the higher physiological strain that ensues in an uncompensable environment (Cheung et al., 2000).

Literature surrounding the practical aspects of HA induction and decay have provided insight into various methods and the many benefits of this method of performance enhancement (Périard et al., 2015). Adaptations in performance tests have been examined in broad sense (i.e., performance vs. exercise capacity), however, no study has examined the effect of HA on specific types of exercise performance tests (Tyler et al., 2016). A recent meta-analysis examined the physiological and perceptual adaptations that occur throughout heat acclimatization induction and decay (Daanen et al., 2018), however, a meta-analysis examining the magnitude of different types of specific performance improvement is absent. Additionally, while several studies have speculated, no previous study has examined the impact of specific moderators on the results observed following HA induction, which could help explain the variability seen in this research. Understanding the magnitude of the performance changes in these tests will be beneficial to athletes, coaches, sports scientists, sport medicine professionals and future researchers who strive to optimize performance and expand the HA literature. Therefore, the aim of this study was two-fold. First, to perform a meta-analysis to examine the magnitude of changes in performance that results from HA in VO_2max_, time to exhaustion, time trial, mean power, and peak power. Second, to determine the impact of moderators on HA performance in these performance tests.

## Materials and Methods

A literature search was conducted using first order search terms (“acclimation,” “acclimatization,” “adaptation”) and second order search terms (“exercise,” “endurance,” “time trial,” “Wingate,” “VO_2max_,” “time to exhaustion”). The search was performed in the following databases: PubMed, Scopus, CINAHL, SportDiscus, Academic Search Premier, and Cochrane Library. The search was conducted February 15, 2019.

### Selection Criteria

**Figure 1:**
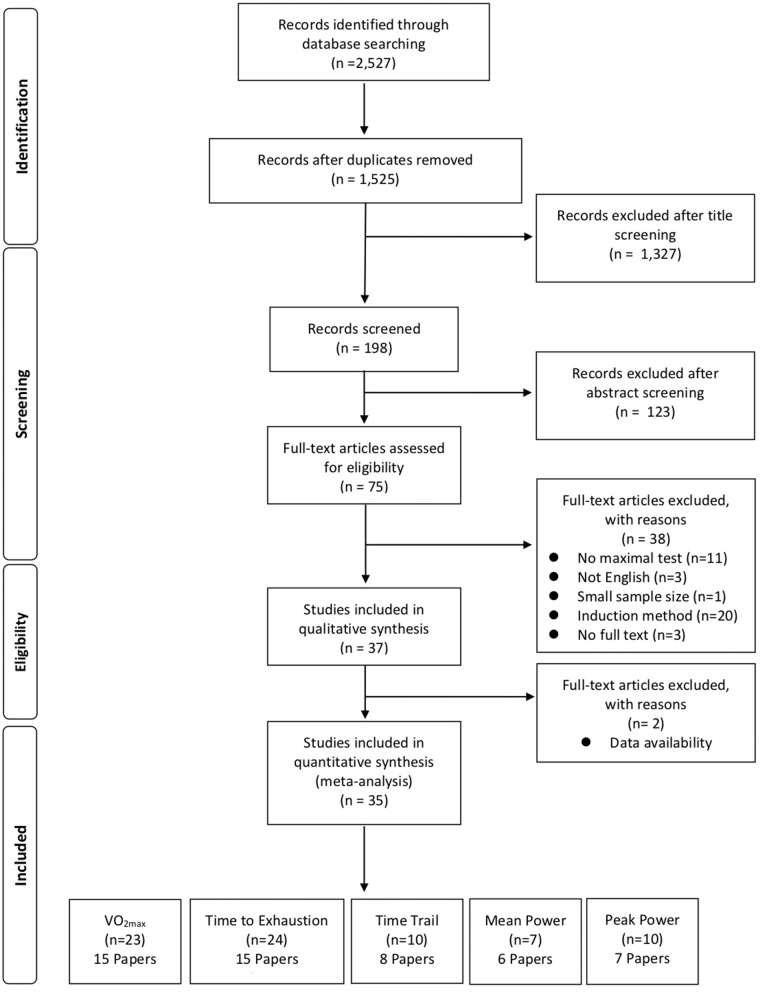
Flow chart summary of the study selection process.

The following search criteria was used to determine the suitability of each paper for this analysis. Figure 1 demonstrates the selection process for this meta-analysis. This meta-analysis only included a study if it met the following requirements:

1. The full-text was available from a peer-reviewed scientific journal in the English language.
2. The study reported a physical performance test outcome for pre and post HA intervention. Cognitive tests were not included in this analysis because the purpose was to assess the effectiveness of HA on various physical performance tests.
3. Only studies that conducted HA were included (not heat acclimatization). The term “acclimatization” was included in the search terms because “acclimation” and “acclimatization” are sometimes used interchangeably in previous research.
4. The mode of exercise during HA occurred on a cycle ergometer or a treadmill. These methods of exercise have high external validity and will be included to control for variability that may be introduced with other methods (i.e., sauna, stair- stepping).
5. The study reported findings from at least four participants to ensure appropriate power for each of the studies.
6. For studies to be included in the time to exhaustion analysis, the baseline test (prior to the start of HA) should be stopped due to volitional fatigue or the laboratory cut-points (such as internal body temperature >40°C), not because of testing time.

### Classification of the Studies

Of the 74 peer-reviewed studies identified, 35 met the inclusion criteria. These studies were categorized by the researchers by type of performance test. Upon review, five types of tests were established: (1) VO_2max_, (2) time to exhaustion, (3) time trial, (4) mean power, and (5) peak power.

### Data Extraction

Studies that involved an additional intervention to HA were included in the analysis only if there was no difference between the control group and the intervention group. In cases that reported differences, only the control group was included.

### Study Quality Assessment

The PEDro scale was not used for the inclusion criteria, however, a quality assessment is included in the results section (Tables 1–5). On this scale, a “high quality” study will score ≥ 7; a “moderate quality” study will score 5 or 6; a “poor quality” study will score ≤ 4 (Maher et al., 2003; Yamato et al., 2017). To assess for publication bias, funnel plots of each performance test can be seen in the supplementary material (Supplementary Figures 1–5).

**Table 1:** VO_2max_ descriptive table.

**References**	***n***	**Induction method^~^**	**Induction length** **(Days)**	**Session duration (min)**	**Exercise intensity^#^**	**Heat index induction**	**Heat index testing**	**Baseline fitness** **(ml·kg^−1^·min^−1^)**	**PEDro**
Horstman and Christensen (1982) A & B	6	Controlled work rate	11	68	Low	54	N/A	51.4	5
	4^*^	Controlled work rate	11	108	Low	54	N/A	47.2	5
King et al. (1985)	10	Controlled work rate	8	90	Low	43	43	46.1	4
Pivarnik et al. (1987)	16	Controlled work rate	6	90	Moderate	43	43	44.2	4
Febbraio et al. (1994)	13	Controlled work rate	7	90	Low	43	39	68.1	7
Aoyagi et al. (1998) A, B, C, & D	6	Controlled work rate	6	60	Low	43	43	47.4	7
	9	Controlled work rate	6	60	Low	43	43	45.1	7
	8	Controlled work rate	6	150	Low	43	43	49.5	7
	8	Controlled work rate	12	150	Low	43	43	48.6	7
Lorenzo et al. (2010)	12	Controlled work rate	10	90	Low	43	39	66.9	6
Chen et al. (2013) A & B	7	Controlled work rate	5	35	Moderate	51	24	53.0	7
	7	Controlled work rate	5	35	Moderate	51	51	53.0	7
Molloy et al. (2013) A & B	9	Controlled work rate	14	30	High	36	22	57.1	6
	7	Controlled work rate	14	30	High	36	22	55.2	6
Keiser et al. (2015)	8	Controlled work rate	10	90	Low	39	39	58.1	7
DiLeo et al. (2016)	10	Controlled work rate	5	90	Low	47	47	50.0	4
Neal et al. (2016a)	10	Isothermal	5	90	Moderate	55	22	63.3	6
Neal et al. (2016b) A & B	8	Isothermal	11	90	Moderate	55	20	56.9	6
	8	Isothermal	11	90	Moderate	55	20	56.9	6
James et al. (2017)	10^∧^	Isothermal	5	90	Moderate	50	37	58.9	6
Rendell et al. (2017)	8	Isothermal	11	90	Moderate	55	22	58.5	6
Willmott et al. (2018) A & B	10	Isothermal	10	60	Moderate	47	21	48.7	6
	10	Isothermal	10	60	Moderate	47	21	48.7	6

~ *Induction method was defined as either “controlled work rate,” which was defined as a constant intensity for a set duration or “isothermal,” which was defined as exercise intensity defined by a pre-determined internal body temperature*.

# *Exercise intensity was defined as either “low,” which was <55% VO_2max_, “moderate,” which was isothermal or 55–70% VO_2max_, and “high,” which was >70% VO_2max_*.

* *Included four females*.

∧ *Included one female*.

**Table 2:** Time to exhaustion descriptive table.

**References**	***n***	**Induction method^~^**	**Induction length** **(Days)**	**Session duration** **(min)**	**Exercise intensity^#^**	**Heat index induction**	**Heat index testing**	**Baseline fitness** **(ml·kg^−1^·min^−1^)**	**PEDro**
Horstman and Christensen (1982)	6	Controlled work rate	11	68	Low	44	44	51.4	5
	4^*^	Controlled work rate	11	108	Low	44	44	47.2	5
Pandolf et al. (1988)	9	Controlled work rate	10	150	Low	55	55	52.9	6
Nielsen et al. (1993) A & B	13	Controlled work rate	10.5	61	Moderate	37	37	59.0	6
Nielsen et al. (1997)	12	Controlled work rate	10	48.3	Low	61	61	62.0	4
Aoyagi et al. (1998)	6	Controlled work rate	6	60	Low	43	43	47.4	7
	9	Controlled work rate	6	60	Low	43	43	45.1	7
Inoue et al. (1999) A, B, & C	5	Controlled work rate	8	90	Low	49	49	47.0	6
	4	Controlled work rate	8	90	Low	49	49	48.0	6
	5	Controlled work rate	8	90	Low	49	49	30.0	6
Garrett et al. (2009)	10	Isothermal	5	90	Moderate	63	45	57.1	4
Burk et al. (2012)	22	Controlled work rate	11	125.3	Moderate	42	42	53.8	4
Chen et al. (2013) A & B	7	Controlled work rate	5	38	N/A	51	24	53.0	7
	7	Controlled work rate	5	38	N/A	51	51	53.0	7
Kaldur et al. (2014)	21	Controlled work rate	10	100	Low	42	42	53.8	4
Oöpik et al. (2014)	20	Controlled work rate	10	100	Low	42	42	53.2	4
Ashley et al. (2015)	10^+^	Controlled work rate	10	120	Low	57	57	33.9	7
	8^+^	Controlled work rate	10	120	Low	57	57	29.2	7
Gibson et al. (2015b) A, B, & C	8	Controlled work rate	10	90	Low	48	42	45.6	6
	8	Isothermal	10	67.4	Moderate	48	42	48.5	6
	8	Isothermal	10	86.1	Moderate	48	42	50.6	6
James et al. (2017)	10^∧^	Isothermal	5	90	Moderate	50	37	58.9	6
Willmott et al. (2018) A & B	10	Isothermal	10	150	Moderate	47	21	48.7	6
	10	Isothermal	10	150	Moderate	47	21	48.7	6

~ *Induction method was defined as either “controlled work rate,” which was defined as a constant intensity for a set duration or “isothermal,” which was defined as exercise intensity defined by a pre-determined internal body temperature*.

# *Exercise intensity was defined as either “low”, which was <55% VO_2max_, “moderate,” which was isothermal or 55–70% VO_2max_, and “high,” which was >70% VO_2max_*.

* *Included four females*.

∧ *Included one female*.

+ *Included five females*.

**Table 3:** Time trial descriptive table.

**References**	***n***	**Induction method^~^**	**Induction length** **(Days)**	**Session duration** **(min)**	**Exercise intensity^#^**	**Heat index induction**	**Heat index testing**	**Baseline fitness** **(ml·kg^−1^·min^−1^)**	**PEDro**
Garrett et al. (2012)	8	Isothermal	5	90	Moderate	61	45	65.0	4
Neal et al. (2016a)	10	Isothermal	5	90	Moderate	55	22	63.3	6
Guy et al. (2016)	8	Controlled work rate	7	N/A	Low	50	50	45.0	7
Lee et al. (2016)	7	Controlled work rate	10	60	Low	41	41	50.7	6
Willmott et al. (2016) A & B	7	Controlled work rate	2	90	Low	45	33	46.1	6
	7	Controlled work rate	4	45	Low	45	33	45.8	6
Wingfield et al. (2016) A & B	10	Controlled work rate	5	90	Low	40	40	44.3	4
	10	Controlled work rate	5	30	High	40	40	41.9	4
James et al. (2017)	10^∧^	Isothermal	5	90	Moderate	50	37	58.9	6
Pethick et al. (2019)	24^+^	Isothermal	5	90	Moderate	40	38	62.3	6

~ *Induction method was defined as either “controlled work rate,” which was defined as a constant intensity for a set duration or “isothermal,” which was defined as exercise intensity defined by a pre-determined internal body temperature*.

# *Exercise intensity was defined as either “low,” which was <55% VO_2max_, “moderate,” which was isothermal or 55–70% VO_2max_, and “high,” which was >70% VO_2max_*.

∧ *Included one female*.

+ *Included two females*.

**Table 4:** Mean power descriptive table.

**References**	***n***	**Induction method^~^**	**Induction length** **(Days)**	**Session duration** **(min)**	**Exercise intensity^#^**	**Heat index induction**	**Heat index testing**	**Baseline fitness** **(ml·kg^−1^·min^−1^)**	**PEDro**
Lorenzo et al. (2010)	12	Controlled work rate	10	90	Low	43	39	66.9	6
Brade et al. (2013)	10	Controlled work rate	5	40	High	45	45	55.3	7
Lee et al. (2016)	7	Controlled work rate	10	60	Low	41	41	50.7	6
Neal et al. (2016a)	10	Isothermal	5	90	Moderate	55	22	63.3	6
Wingfield et al. (2016) A & B	10	Controlled work rate	5	90	Low	40	40	44.3	4
	10	Controlled work rate	5	30	High	40	40	41.9	4
Duvnjak-Zaknich et al. (2018)	8	Controlled work rate	8	41	N/A	45	46	54.3	7

~ *Induction method was defined as either “controlled work rate,” which was defined as a constant intensity for a set duration or “isothermal,” which was defined as exercise intensity defined by a pre-determined internal body temperature*.

# *Exercise intensity was defined as either “low,” which was <55% VO_2max_, “moderate,” which was isothermal or 55–70% VO_2max_, and “high,” which was >70% VO_2max_*.

**Table 5:** Peak power descriptive table.

**References**	***n***	**Induction method^~^**	**Induction length (Days)**	**Session duration** **(min)**	**Exercise intensity^#^**	**Heat Index induction**	**Heat index testing**	**Baseline** **fitness** **(ml·kg^−1^·min^−1^)**	**PEDro**
(Castle et al., 2011) A & B	8	Controlled work rate	10	60	Low	37	38	43.3	6
	8	Controlled work rate	10	60	Low	37	38	43.3	6
Brade et al. (2013)	10	Controlled work rate	5	40	High	45	45	55.3	7
Keiser et al. (2015)	8	Controlled work rate	10	90	Low	30	39	61.2	7
Neal et al. (2016a)	10	Isothermal	5	90	Moderate	55	22	63.3	6
(Neal et al., 2016b) A & B	8	Isothermal	11	90	Moderate	55	20	56.9	6
	8	Isothermal	11	90	Moderate	55	20	56.9	6
Rendell et al. (2017)	8	Isothermal	11	90	Moderate	55	20	N/A	6
Willmott et al. (2018) A & B	10	Isothermal	10	60	Moderate	47	43	48.7	6
	10	Isothermal	10	60	Moderate	47	43	48.7	6

~ *Induction method was defined as either “controlled work rate,” which was defined as a constant intensity for a set duration or “isothermal,” which was defined as exercise intensity defined by a pre-determined internal body temperature*.

# *Exercise intensity was defined as either “low,” which was <55% VO_2max_, “moderate,” which was isothermal or 55–70% VO_2max_, and “high,” which was >70% VO_2max_*.

### Data Analysis

This meta-analysis was performed in Comprehensive Meta-Analysis software (version 2.2.064, Biostat company, Englewood, NJ, USA). Studies included in this analysis reported data to determine the changes within group, between pre and post HA. In the event that correlation values were not available, the lowest available correlation value for that test was utilized to calculate the effect size. Data are reported as mean (M), standard deviation (SD) mean difference (MD), Hedge's g effect size (ES), and 95% confidence intervals (95% CI). Statistical significance was set to *p* < 0.05, a priori.

To determine the effects of moderators on outcomes in performance, a meta-regression was performed. A separate meta-regression was performed for each performance test type and moderators were entered separately as individual models in the analysis. Moderators that were considered for all test types in this analysis included: fitness level, induction length, session duration, exercise intensity, induction method, heat index of induction, and heat index of testing. For the “fitness level” moderator, baseline (prior to HA induction) VO_2max_ levels were utilized. Induction length was entered as a continuous variable as the number of days utilized for HA induction. Session duration was entered as a continuous variable as the total number of minutes for each session throughout HA induction. In cases that involved a progressive duration protocol, the average duration time was entered. Exercise intensity was defined as “low,” “moderate,” and “high,” with “low” being defined as <55% VO_2max_, “moderate” between 55 and 70% VO_2max_ or isothermal, and “high” being greater that 70% VO_2max_ and “low” was set as the reference group. Induction method was defined as “controlled work rate” or “isothermal,” with “controlled work rate” referring to an intensity that was set by the investigator throughout HA induction and “isothermal,” referring to HA sessions adjusting the exercise intensity seeking to maintain the internal body temperature to a pre-determined criteria (typically 38.5°C). For this moderator, “controlled work rate” was set as the reference group. Environmental conditions of both induction and testing were measured as heat index and were calculated from reported environmental conditions. Tests were included if they were performed in hot (≥25°C) or thermoneutral (<25°C) environmental conditions. Three of the time to exhaustion tests were performed in thermoneutral environments. Twenty of the time to exhaustion tests were performed in a hot environment. Two studies did not report the environmental conditions of the testing sessions. In terms of mean power, one study conducted the performance test in a thermoneutral environmental condition. Of the VO_2max_ tests, nine were performed in thermoneutral conditions and 12 were performed in hot conditions (two not reported). For time trial performance, distance was used as a moderator to see the impact of HA induction on various time trial results.

## Results

### Search Results

In total, 2,527 articles were found. Of those articles, 35 met the inclusion/exclusion criteria with 23 studies that assessed VO_2max_ (*n* = 204), 24 studies that assessed time to exhaustion (*n* = 232), 10 studies that performed time trials (*n* = 101), 7 studies that assessed mean power (*n* = 67), and 10 studies that assessed peak power (*n* = 88). The fitness level prior to the start of HA was reported (M ± SD; VO_2max_, 53.7 ± 6.8 ml·kg^−1^·min^−1^; time to exhaustion 49.2 ± 8.1 ml·kg^−1^·min^−1^; time trial, 52.3 ± 9.0 ml·kg^−1^·min^−1^; mean power, 53.8 ± 9.2 ml·kg^−1^·min^−1^; peak power, 53.1 ± 7.4 ml·kg^−1^·min^−1^. Most studies investigated only males, however, some of the studies reported female data. The quality of the included manuscripts was assessed by two readers using the PEDro scale (M ± SD; 5.6 ± 1.1). The nature of HA induction does not allow for blinding of the participants. Descriptive information about each of the studies for each performance test type can be seen in Tables 1–5.

### Impact of HA on Various Performance Tests

**Figure 2:**
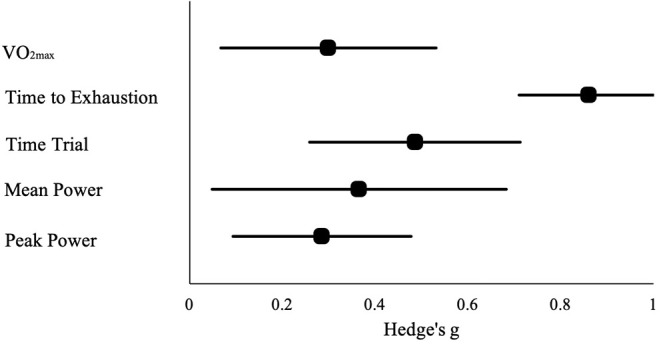
Magnitude of performance changes observed in each performance test following heat acclimation. Data are presented as Hedge's g and 95% confidence intervals.

**Figure 3:**
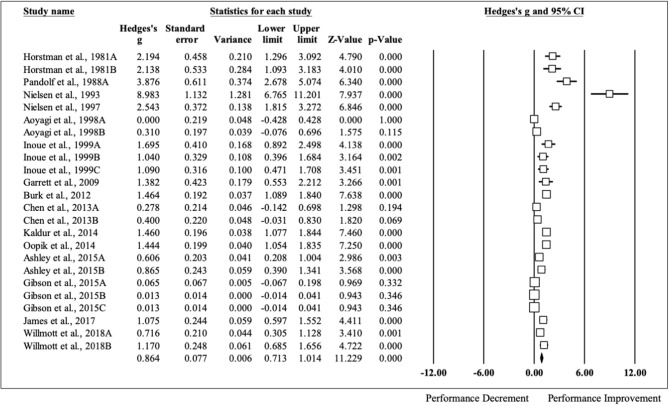
Time to exhaustion forest plot. Data are presented as Hedge's g and 95% confidence intervals.

**Figure 4:**
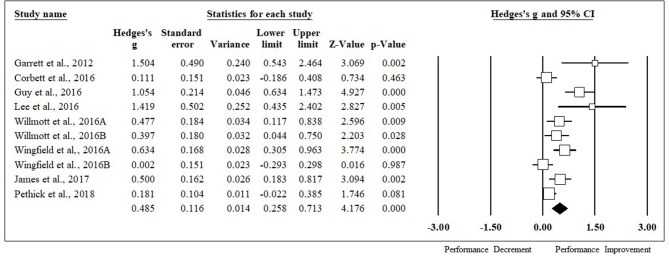
Time trial forest plot. Data are presented as Hedge's g and 95% confidence intervals.

**Figure 5:**
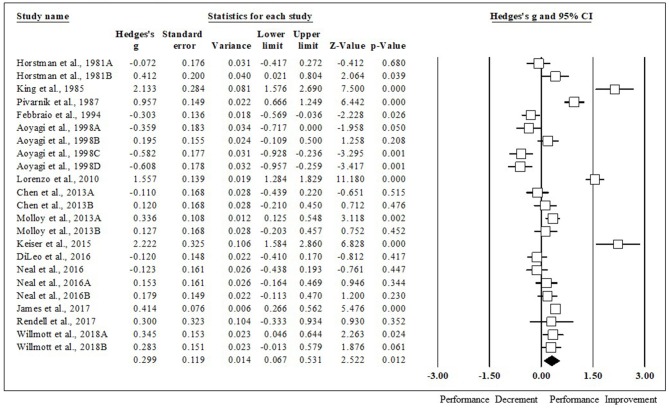
VO_2max_ forest plot. Data are presented as Hedge's g and 95% confidence intervals.

**Figure 6:**
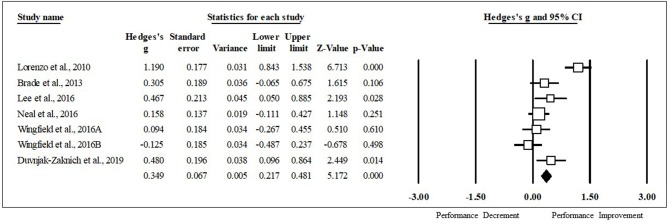
Mean power forest plot. Data are presented as Hedge's g and 95% confidence intervals.

**Figure 7:**
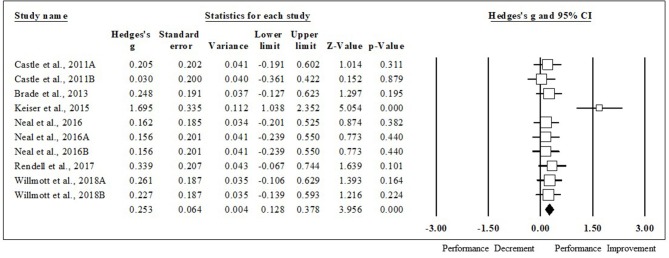
Peak power forest plot. Data are presented as Hedge's g and 95% confidence intervals.

HA had a positive impact on performance, regardless of testing type (ES [95% CI]; = 0.53 [0.44, 0.63], *p* < 0.001). HA induced positive adaptions for each performance test [MD [95% CI]; time to exhaustion, 144.30 s [128.30, 160.31], *p* < 0.001; time trial, −45.60 s [−22.80, −68.40], *p* < 0.001; mean power 12 W [2.08, 22.37], *p* = 0.02; VO_2max_, 1.32 ml·kg^−1^·min^−1^ [0.20, 2.43], *p* = 0.02; peak power 15 W [8.90, 21.09], *p* < 0.001). The magnitude of change following HA induction was analyzed, with time to exhaustion demonstrating the largest performance enhancement, followed by time trial, mean power, VO_2max_, and peak power (ES [95% CI]; time to exhaustion, 0.86 [0.71, 1.01], *p* < 0.001; time trial, 0.49 [0.26, 0.71], *p* < 0.001; mean power, 0.37 [0.05, 0.68], *p* < 0.001; VO_2max_, 0.30 [0.07, 0.53], *p* = 0.012; peak power, 0.29 [0.09, 0.48], *p* < 0.001) (Figure 2). ES for each of the studies for time to exhaustion (Figure 3), time trial (Figure 4), mean power (Figure 5), VO_2max_ (Figure 6), and peak power (Figure 7) were demonstrated.

### Time to Exhaustion Meta-Regression

**Figure 8:**
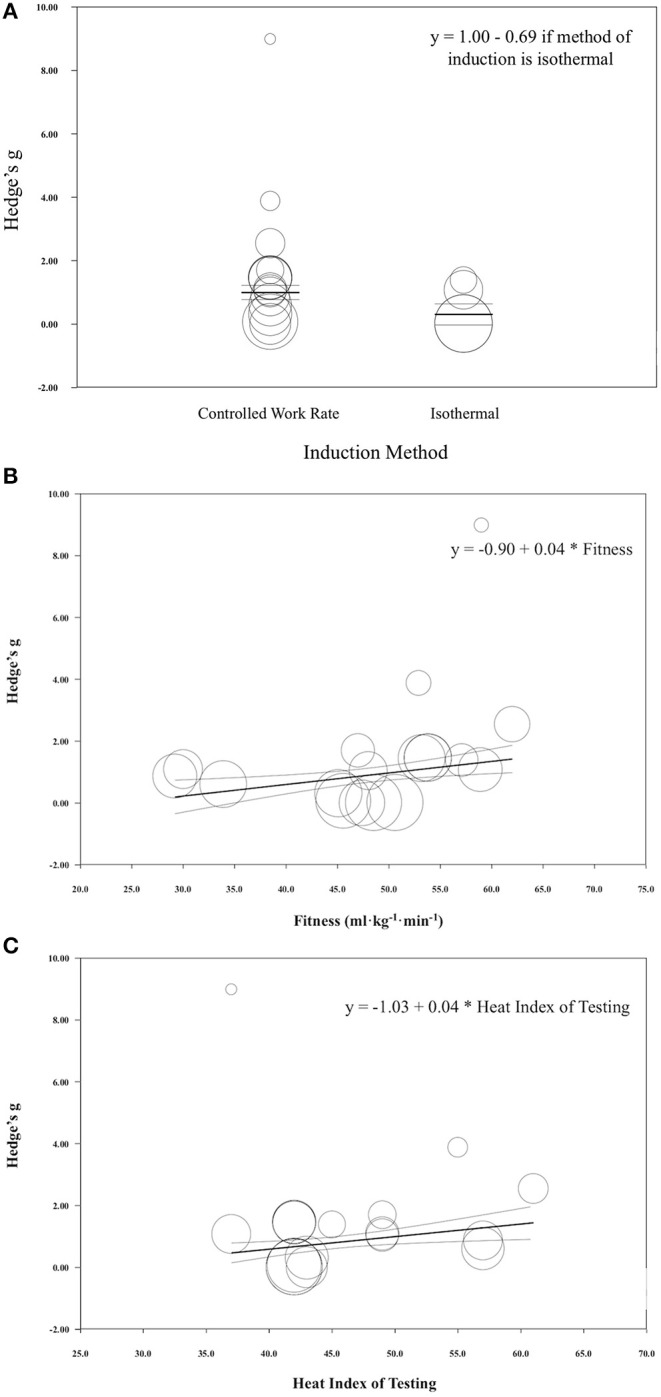
**(A)** Regression of Hedge's g on induction method for time to exhaustion exercise performance following heat acclimation. Solid black bars represent the mean Hedge's g. Each circle represents individual studies. The size of the circle represents the weight of that study that was applied in the analysis. Smaller circles indicate lower weight and larger circles indicate higher weight. **(B)** Regression of Hedge's g on fitness level for time to exhaustion exercise performance following heat acclimation. Solid black bars represent the mean Hedge's g. Each circle represents individual studies. The size of the circle represents the weight of that study that was applied in the analysis. Smaller circles indicate lower weight and larger circles indicate higher weight. **(C)** Regression of Hedge's g on heat index for time to exhaustion exercise performance following heat acclimation. Solid black bars represent the mean Hedge's g. Each circle represents individual studies. The size of the circle represents the weight of that study that was applied in the analysis. Smaller circles indicate lower weight and larger circles indicate higher weight.

When all of the covariates were analyzed as individual models, induction method significantly impacted the magnitude of change seen in time to exhaustion following HA induction (coefficient [95% CI]; −0.69 [−1.01, −0.37], *r*^2^ = 0.26, *p* < 0.001) (Figure 8A). Fitness level also significantly impacted the change seen in time to exhaustion, however, no variance in the results was explained by this model (coefficient [95% CI]; 0.04 [0.02, 0.06], *r*^2^ = 0.00, *p* < 0.001) (Figure 8B). The heat index of testing also explained some of the variance seen in this test time (coefficient [95% CI]; 0.04 [0.02, 0.07], *r*^2^ = 0.18, *p* < 0.001) (Figure 8C). All other covariates did not significantly impact the magnitude of change seen in time to exhaustion following HA (coefficient [95% CI]; induction length, 0.04 [−0.04, 0.13], *p* = 0.34; session duration, 0.01 [−0.00, 0.02], *p* = 0.08; exercise intensity, −0.27 [−0.59, 0.05], *p* = 0.10; heat index of induction, 0.01 [−0.01, 0.04], *p* = 0.30). Of the 19 times to exhaustion tests in a hot environment, 18 saw improved performance, while one saw no changes in performance.

### Time Trial Meta-Regression

When all of the covariates were entered as individual models, they did not significantly impact the magnitude of change seen from HA, however, high intensity training was approaching significance and this variable explained 24% of the variance seen in this type of performance test (coefficient [95% CI]; fitness level, 0.00 [−0.02, 0.03], *p* = 0.91; induction length, 0.08 [−0.07, 0.23], *p* = 0.28; session duration, 0.00 [−0.01, 0.01], *p* = 0.40; high intensity, −0.58 [−1.17, 0.02], *p* = 0.06; moderate intensity, −0.23 [−0.65, 0.18], *p* = 0.26; induction method, −0.08 [−0.53, 0.37], *p* = 0.74; heat index of induction, 0.01 [−0.02, 0.05], *p* = 0.45; heat index of testing, 0.03 [−0.01, 0.06], *p* = 0.16). In terms of environmental conditions, time trial performance was improved in all studies, however, only one study investigated a time trial in thermoneutral conditions.

### Mean Power Meta-Regression

**Figure 9:**
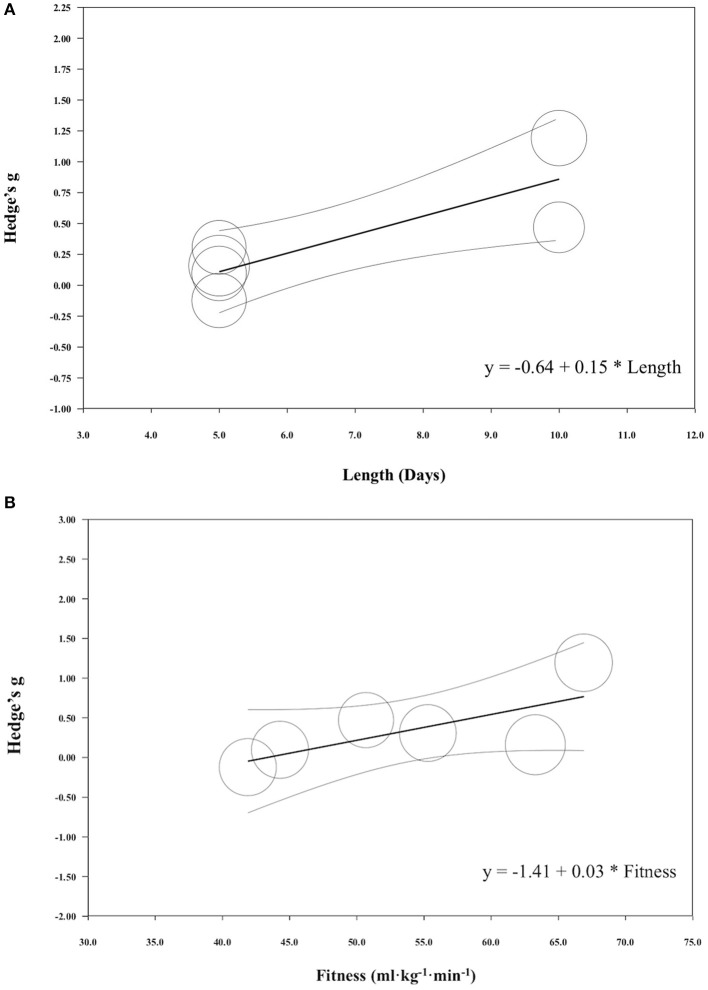
**(A)** Regression of Hedge's g on induction length for mean power exercise performance following heat acclimation. Solid black bars represent the mean Hedge's g. Each circle represents individual studies. The size of the circle represents the weight of that study that was applied in the analysis. Smaller circles indicate lower weight and larger circles indicate higher weight. **(B)** Regression of Hedge's g on fitness level for mean power exercise performance following heat acclimation. Solid black bars represent the mean Hedge's g. Each circle represents individual studies. The size of the circle represents the weight of that study that was applied in the analysis. Smaller circles indicate lower weight and larger circles indicate higher weight.

When all of the covariates were run as individual models, induction length significantly impacted the magnitude of change seen in mean power following HA induction (coefficient [95% CI]; induction length 0.15 [0.05, 0.25], *r*^2^ = 0.75 *p* = 0.002) (Figure 9A). All other covariates did not significantly impact the magnitude of change seen in mean power from HA, however, fitness level was approaching significance and this variable explained 30% of the variance observed in this performance test (coefficient [95% CI]; fitness level, 0.03 [−0.001, 0.07], *p* = 0.06 (Figure 9B); session duration, 0.01 [−0.01, 0.02], *p* = 0.36; high intensity, −0.43 [−1.41, 0.41], *p* = 0.28; moderate intensity, −0.43 [−1.55, 0.69], *p* = 0.45; induction method, −0.23 [−1.33, 0.87], *p* = 0.68; heat index of induction, 0.00 [−0.08, 0.08], *p* = 0.94; heat index of testing, 0.01 [−0.05, 0.06], *p* = 0.78). One study that conducted a performance test in a thermoneutral environmental condition observed improved performance, while five out of the six tests that were performed in the heat saw improvements.

### VO_2max_ Meta-Regression

When all of the covariates were run as individual models, they did not significantly impact the magnitude of change seen in VO_2max_ from HA (coefficient [95% CI]; fitness level, −0.01 [−0.02, 0.05], *p* = 0.48; induction method, −0.14 [−0.69, 0.41], *p* = 0.62; session duration, 0.00 [−0.01, 0.01], *p* = 0.64; induction length, 0.03 [−0.06, 0.11], *p* = 0.54; high intensity, −0.44 [−1.26, 0.37], *p* = 0.29; moderate intensity, −0.25 [−0.82, 0.33], *p* = 0.41; heat index of induction, −0.03 [−0.07, 0.02], *p* = 0.20; heat index of testing, −0.01 [−0.02, 0.03], *p* = 0.59). Of the nine thermoneutral VO_2max_ tests, eight observed improvements in performance following HA induction. Of the 12 in hot tests, seven observed performance improvements following HA induction.

### Peak Power Meta-Regression

When all of the covariates were run as individual models, they did not significantly impact the magnitude of change seen in peak power from HA (coefficient [95% CI]; fitness level, 0.02 [−0.01, 0.05], *p* = 0.22; induction length, 0.01 [−0.08, 0.10], *p* = 0.77; session duration, 0.00 [−0.01, 0.02], *p* = 0.40; high intensity, −0.24 [−1.02, 0.54], *p* = 0.54; moderate intensity, −0.27 [−0.77, 0.22], *p* = 0.28; induction method, −0.19 [−0.61, 0.22], *p* = 0.36; heat index of induction, −0.02 [−0.04, 0.003], *p* = 0.09; heat index of testing, 0.01 [−0.01, 0.03], *p* = 0.56). Peak power performance improved in all tests, regardless of testing environmental conditions (thermoneutral, *n* = 4; hot, *n* = 6).

## Discussion

The largest performance improvement was observed in time to exhaustion with an average improvement of 144.30 s. Tyler et al. demonstrated in a meta-analysis that exercise capacity improved on average 23% (Tyler et al., 2016). Additionally, internal body temperature decreases an average of 0.31°C and heart rate lowers 12 bpm following HA (Tyler et al., 2016). In the present analysis, time to exhaustion tests were terminated when either participants reached their maximal efforts or heart rate/internal body temperature exceeded the lab safety criteria. One possible mechanism that could explain the improvements seen in time to exhaustion include lower internal body temperature (at baseline and during exercise) and heart rate that occur over the course of HA. The second largest magnitude of improvement was observed in time trials (MD, −45.6 s), followed by mean power (MD, 12 W), VO_2max_ (MD, 1.32 ml·kg^−1^·min^−1^), and peak power (MD, 15 W). Previous research demonstrated a 7% improvement in performance tests following HA (Tyler et al., 2016). These performance improvements following HA were most likely due to increases in maximal cardiac output, lactate threshold and plasma volume, lowered skin temperature and a larger core-to-skin gradient as seen in previous research (Périard et al., 2015). However, VO_2max_ might be impacted through improved fitness induced by exercise training alone compared to HA specifically (Brooks et al., 2015). Finally, peak power is not specifically a measurement of aerobic performance, thus, might not be impacted as substantially as other tests from HA.

One potential moderator that could explain some of the variance between studies that could not be accounted for in this analysis is the number of days of rest following HA before testing. There were a wide variety of reporting methods for the metric that does not allow for certainty in this analysis. For example, many manuscripts reported completing the performance test “within x number of days,” meaning some participants may have completed the test the day after HA induction and other participants may have completed the test on day x after the end of HA induction. In general studies reported completing the tests anywhere from one to seven days following HA induction. A recent paper by Daanen et al. demonstrated that internal body temperature was lowered three and seven days following HA induction compared to the day immediately following HA induction. Thus, leading one to believe that performance adaptations may also be improved with a few days of recovery following HA induction, however, future research is needed (Daanen et al., 2011). Another meta-analysis has extensively examined the timeline of HA decay and concluded that internal body temperature and heart rate responses typically decay at a rate of 2.5% per day (Daanen et al., 2018). The importance of recovery has even been examined and reported when seeking optimal training improvements in a thermoneutral environment and 96 h of rest following training was suggested (Waldron et al., 2019). The results from both of these manuscripts point to the importance of finding the appropriate balance between recovery and acclimation decay for optimal performance results.

While differentiating the performance outcomes between HA and training alone is of importance, the current analysis did not examine these differences. Of the manuscripts included in this analysis, only 11 included a control group (time trial, *n* = 5; time to exhaustion, *n* = 4; VO_2max_, *n* = 6; peak power, *n* = 3; mean power, *n* = 2). HA appears to improve time trial performance compared to controls (Guy et al., 2016; Lee et al., 2016; Willmott et al., 2016; James et al., 2017). One short term HA protocol (4–6 days) did not elicit statistically significant improvements in time trial performance compared to a control group, however, moderate to large effect sizes were reported (Willmott et al., 2016). Time to exhaustion improved with HA in all studies, but not with control groups (Nielsen et al., 1993; Chen et al., 2013; James et al., 2017; Willmott et al., 2018). In terms of VO_2max_, the performance differences between HA and training are unclear, as some studies reported differences between the groups and other did not (Lorenzo et al., 2010; Chen et al., 2013; Keiser et al., 2015; James et al., 2017; Rendell et al., 2017; Willmott et al., 2018). Peak power may improve with HA compared to training alone, however, the results are unclear and future research is needed (Keiser et al., 2015; Rendell et al., 2017; Willmott et al., 2018). Both studies that assessed mean power demonstrated improved performance benefits from HA compared to a control group (Lorenzo et al., 2010; Lee et al., 2016). To determine the true performance changes of HA compared to training alone, future studies should aim to include a control group within their study design.

### Time to Exhaustion

As previous research has clearly established, HA is an effective strategy to improve time to exhaustion and this was evident in the current meta-analysis, as no study reported decrements. The study that observed the largest performance improvement (ES = 8.98) took place with the participants who held the highest VO_2max_ (62.0 ml·kg^−1^·min^−1^), hypothetically giving them a higher training ceiling (Nielsen et al., 1993; Chen et al., 2013; James et al., 2017; Willmott et al., 2018). The HA induction took place over the course of 10 days for ~48 min per session at a low exercise intensity (120 beats per minute; ~45% VO_2max_) and in the most extreme environmental conditions (ambient temperature, 35.4 ± 0.05°C; relative humidity, 87.2 ± 0.04%) of any study included in this analysis (Nielsen et al., 1997). Pandolf et al. also observed large improvements in time to exhaustion following HA (ES = 3.88) with a controlled work rate exercise intensity for 150 min over 10 days in relatively fit, middle-age individuals (VO_2max_ = 52.9 ml·kg^−1^·min^−1^) (Pandolf et al., 1988). The purpose of this particular research was to examine differences in young and middle age males over the course of HA who were matched for several morphological factors and the magnitude of performance time change was much larger for the younger group than the middle age group, due to the younger group reaching exhaustion much sooner than the middle age group at the beginning of HA, however, the middle-age group was not included in this analysis since their baseline test did not meet the inclusion criteria (Pandolf et al., 1988). The authors of this study hypothesized that the higher training volume of the middle aged men explained their thermoregulatory advantage at the beginning of HA, as they reported running on average 20 more miles per week than the younger men, pointing to the importance of previous training for improved thermoregulation capabilities (Pandolf et al., 1988). Despite this difference, HA induction successfully allowed the younger men to reach the same thermoregulatory capacity as middle aged men (Pandolf et al., 1988).

Two studies included in this meta-analysis did not observe any time to exhaustion performance improvements following HA induction (Pandolf et al., 1988; Aoyagi et al., 1998). One potential explanation of these findings in one of these studies is the low exercise intensity of the test (walk at 1.34 m·s^−1^ to exhaustion), allowing participants to complete the test to completion before HA induction ensued (Aoyagi et al., 1998). Similarly, the other group in the Pandolf et al. study was able to tolerate the test well on the first day of HA, most likely due to their training history (Pandolf et al., 1988).

Of the moderators entered into the meta-regression, induction method and fitness level appear to explain some of the variance seen in this type of performance test following HA. Controlled work rate exercise intensity during HA appears to hold a slight advantage over isothermal (controlled work rate ES = 1.00; isothermal ES = 0.31). One possible mechanism to explain this finding is the potential increase in area under the heating curve with controlled work rate exercise intensity during HA, as the isothermal method might actually lead to a lower overall thermal load since the exercise is adjusted to maintain a temperature of 38.5°C (Bardis et al., 2013).

While recent evidence suggests that peak internal body temperatures of 39°C are not more advantageous than the traditional isothermal temperature of 38.5°C (Gibson et al., 2015b, 2019), there are perhaps greater improvements with increased levels of hyperthermia (>39.0°C), especially in elite level athletes. Data from the Union Cycliste Internationale Road Cycling World Championship demonstrated the capability of elite level athletes to tolerate internal body temperatures well above what is often reported in the HA literature (as high as 41.5°C), however, future research is needed in this area. An increased thermal load has the potential to drive HA through several mechanisms, including, an increased cardiac response, skin temperature, and sweat rate (Shibasaki et al., 2006; Périard et al., 2016). While increased internal body temperature has the potential to elicit greater HA adaptations, a valid measure of internal body temperature (ingestible thermistor or rectal temperature) and professionals trained in recognizing and treating exertional heat illness is needed when intentionally inducing HA in this way to ensure athlete safety.

Fitness level also appeared to impact the results seen in this performance test, as studies with higher starting VO_2max_ values appeared to have greater improvement in this type of performance test. For example, an individual with a VO_2max_ of 60 ml·kg^−1^·min^−1^ (predicted ES = 1.50) is likely to achieve a larger magnitude of performance changes following HA compared to an individual with 40 ml·kg^−1^·min^−1^ (predicted ES = 0.7). Because of the stimulation of sweating and skin blow flow (Piwonka et al., 1965), improved evaporative cooling (Gisolfi and Robinson, 1969), greater cardiac stability (Strydom and Williams, 1969), changes in fluid dynamics (Senay, 1979), earlier onset of sweating (Baum et al., 1976; Nadel, 1979), and greater sweat sensitivity (Wells et al., 1980), aerobically trained individuals appear to show partial HA benefits (Armstrong et al., 1987).

### Time Trial

Time trial performance is arguably the most applicable in the sport setting and every study included in this meta-analysis demonstrated faster times following HA induction. Time trial was improved by −0.76 min on average. Garrett et al. saw the largest improvement in time trial performance (ES = 1.50) following HA using the isothermal method for 90 min over 5 days in participants holding the highest VO_2max_ (65.0 ml·kg^−1^·min^−1^) in the most extreme environmental conditions (ambient temperature, 39.5°C; relative humidity, 60%). A previous review by Periard et al. demonstrated aerobically fit individuals can develop adaptations to HA rapidly (Périard et al., 2015). Lee et al. also demonstrated large improvements following HA induction (ES = 1.42) (Lee et al., 2016). The HA induction took place with a controlled work rate for 60 min over the course of 10 days with relatively fit individuals (VO_2max_ = 50.7 ml·kg^−1^·min^−1^). Willmott et al. showed the smallest improvements following HA with 30 min of exercise at a high intensity controlled work rate for 5 days in 32°C and 60% relative humidity (ES = 0.002). Previous research suggested 60–120 min of exercise duration to induce optimal adaptations following HA, therefore, 30 min of exercise for each session in this study might not be enough to elicit optimal adaptations (Sawka et al., 2011).

Of the moderators entered into the meta-regression, exercise intensity might explain some of the variance (24%) seen in this type of performance test following HA even though it was not significant. Low intensity exercise induced large adaptations in time trial performance (High intensity, ES = 0.00; Moderate intensity; ES = 0.35; Low intensity, ES = 0.58), which could be due to lower levels of fatigue from HA that might be seen with high or moderate exercise intensity.

### VO_2max_

It has been well-established that fitness level contributes substantially to someone's ability to thermoregulate and that individuals with higher fitness levels already demonstrate some physiological parameters of HA (Pandolf et al., 1977). One interesting phenomenon that is evident from this meta-analysis is that there are also improvements of VO_2max_ following HA induction. Keiser et al. observed a 9.6% improvement in VO_2max_ following 10 days of 90 min low intensity HA sessions (Keiser et al., 2015). Lorenzo et al. also demonstrated large improvements in VO_2max_ following 10 days of 90 min, low intensity HA sessions (MD ± SD, −4.5 ± −0.5 ml·kg^−1^·min^−1^) (Lorenzo et al., 2010). VO_2max_ was lower following HA in eight studies, unlike time to exhaustion and time trial performance tests that did not demonstrate any negative outcomes following HA.

There are several factors that could help explain these negative findings, including the fatigue, training impulse, and the participant's starting fitness levels. Similar to any novel training, HA introduces new stress to the body and can lead to fatigue. Daanen et al. recently demonstrated further performance improvements following HA when a break was initiated at the cessation of induction prior to the performance test (Daanen et al., 2011), allowing the participants time to recover and reap the full benefits of HA. Aoyagi et al. demonstrated the largest decrement in VO_2max_ following HA (MD ± SD, −1.4 ± −0.4 ml·kg^−1^·min^−1^), that involved 150 min (longest exercise duration) of 12 days of HA with only one rest day (Aoyagi et al., 1998). Similarly, Febbraio et al. saw decrements in VO_2max_ following HA induction (MD ± SD, −1.5 ± −0.6 ml·kg^−1^·min^−1^) in highly fit participants, however, the test took place within 24 h of the final HA session, which might not have allowed the full adaptations to take place (Febbraio et al., 1994). There were no moderators that largely impacted the magnitude of changes seen in VO_2max_.

### Power

Power is another critical performance measurement that can be applicable in sport settings. In this meta-analysis, mean power and peak power were analyzed. Mean power was improved by 12 W, on average. Lorenzo et al., reported the largest improvements following HA (Lorenzo and Minson, 2010). HA induction took place with 90 min of low intensity, controlled work rate for 10 days at 40°C and 30% relative humidity. Duvnjak-Zaknich et al. also showed large improvements following HA in mean power (ES = 0.480), in which the HA induction took place with 41 min of controlled work rate exercise for 8 days at 35°C and 60% relative humidity (Duvnjak-Zaknich et al., 2018). Lee et al. also showed large improvements in mean power (ES = 0.467). However, Wingfield et al. demonstrated negative mean power result following 30 min of high intensity controlled work rate HA for 5 days and the smallest improvement following 90 min of low intensity control work rate for 5 days in 33°C and 60% relative humidity (Wingfield et al., 2016). The studies showing larger improvements achieved longer length of HA induction. In addition to this point, Wingfield et al. measured mean power during five times of 6 s sprints, while other studies performed mean power during aerobic exercise test, such as 60 min exercise. HA induction could be more beneficial to improve mean power during aerobic exercise following longer duration of induction length.

Induction length explained 75% of the variance seen in power output following HA. For example, when HA induction length was 10 days, the predicted magnitude of change was ES = 0.86 and when it was 5 days, the predicted magnitude of change was only ES = 0.11. This finding is in line with original research which pointed to the full adaptations of HA taking 10 days (Armstrong and Maresh, 1991). However, these findings should be interpreted with caution, as there were only seven studies included in this analysis and other variables, such as the participant's previous training history, were not accounted for and could contribute to this variability. In fact, fitness level was approaching statistical significance in the regression model and may contribute to the variability with increased statistical power.

Peak power was improved 15 W, on average. Keiser et al. showed the largest improvements (ES = 1.695) which took place with 90 min of low intensity exercise for 10 days in 33°C and 39% relative humidity, while other studies showed smaller improvements following HA (ES = 0.030–0.339). Peak power was measured during a graded exercise test, repeated short sprint test, and longer duration exercise. There were no moderators that significantly impacted the results seen in peak power, most likely due to this type of test not directly measuring aerobic capacity, but more likely anaerobic capacity.

### Limitations

While the goal of this meta-analysis was to provide an overview of various performance tests, this meta-analysis was not without limitations. One limitation of this meta-analysis was that some papers did not report correlations which was necessary to calculate ES in the statistical software. In this case, the lowest correlation value was used to achieve the most conservative outcomes. Even though females were included in this analysis, it is unclear if the current findings can be extrapolated to this population due to the variety of or lack of control over menstrual cycle status. For example, one study did not control for menstrual cycle status and simply reported that the findings were not different when females were excluded from the analysis (Pethick et al., 2019). Another study reported completing pre-tests and HA during the follicular phase of the menstrual cycle and post-tests during the luteal phase (James et al., 2017). Still, some did not report information about menstrual cycle status (Horstman and Christensen, 1982; Ashley et al., 2015). A further limitation of this meta-analysis was that each type of performance test had slightly different testing methods. For example, the distance of the time trial was not the same among the studies but were still categorized as a time trial. The moderator analysis may help with the interpretation of these results. While the physiological mechanisms behind power, endurance, and sprint tests cannot be understated, there were not enough peak power and mean power studies to utilize these categories as moderators in the current analysis. Another limitation was when data needed to calculate effect size was not reported in text or tables and it was demonstrated in figures, the data was estimated using a ruler. Additionally, while all studies reported the use of internal body temperature assessment, very few reported the actual internal body temperature data during HA induction and this meta-analysis could not include this information as a moderator. However, a previous review indicated an increased internal body temperature during HA is a critical factor to induce adaptations (Périard et al., 2015). Future research should ensure that the internal body temperature data during the HA sessions are reported.

## Conclusions and Practical Application

A wide range of HA induction protocols have been investigated in this meta-analysis. The largest performance improvement was observed in time to exhaustion followed by time trial, mean power, VO_2max_, and peak power following HA. The results observed in these performance tests were each impacted differently by specific moderators. Performance enhancements were greater in time to exhaustion tests when a controlled work rate method was utilized for HA and when the participants of these studies began the HA with higher baseline fitness levels, as indicated by VO_2max_. Time trial results were improved if the HA induction involved low exercise intensity, which could be related to the participants in these studies not experiencing fatigue from high intensity HA. Longer HA induction (i.e., 10 days) appeared to elicit greater adaptations in mean power than short HA induction (i.e., 5 days). Sport scientists and researchers can use the findings from this meta-analysis to customize the design of HA induction protocols to maximize the adaptations of specific performance tests.

## Author Contributions

CB and YS developed the idea of the meta-analysis, completed the analysis, and created the figures. CB, YS, and LF worked to review and code all of the manuscripts. LF created the tables. DC verified the manuscript review and analytical methods. All authors reviewed and edited the manuscript.

### Conflict of Interest

DC is employed at the Korey Stringer Institute at the University of Connecticut. He has received numerous grants (over 20 in the past 3 years), but none connected to this publication. He has also received consulting fees or honorariums from Clif Bar, Sports Innovation Lab, and the National Football League. He has received payment for lectures from Gatorade. He has been an expert witness in over 40 cases. He also received royalties from Jones and Bartlett Publishers, LWW, Springer, and Up-to-Date for two published books. The remaining authors declare that the research was conducted in the absence of any commercial or financial relationships that could be construed as a potential conflict of interest.
