# Supplementary figures and images for: Performance Changes Following Heat Acclimation and the Factors That Influence These Changes: Meta-Analysis and Meta-Regression
Source: Front Physiol. 2019 Nov 27;10:1448. doi: 10.3389/fphys.2019.01448 (PMC6890862; doi:10.3389/fphys.2019.01448)

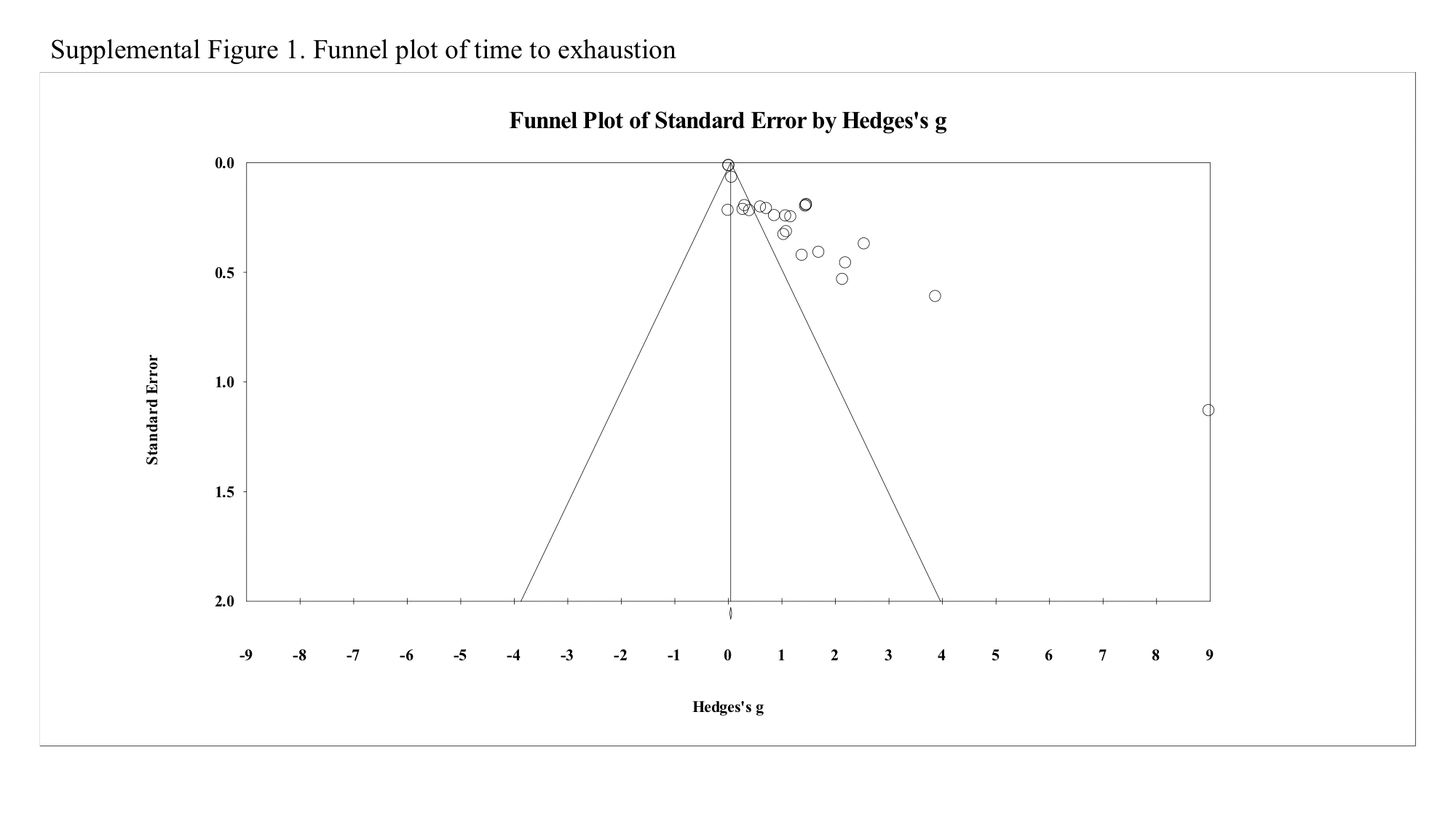

Supplement: Supplementary file 1 [file Image_1.TIFF]

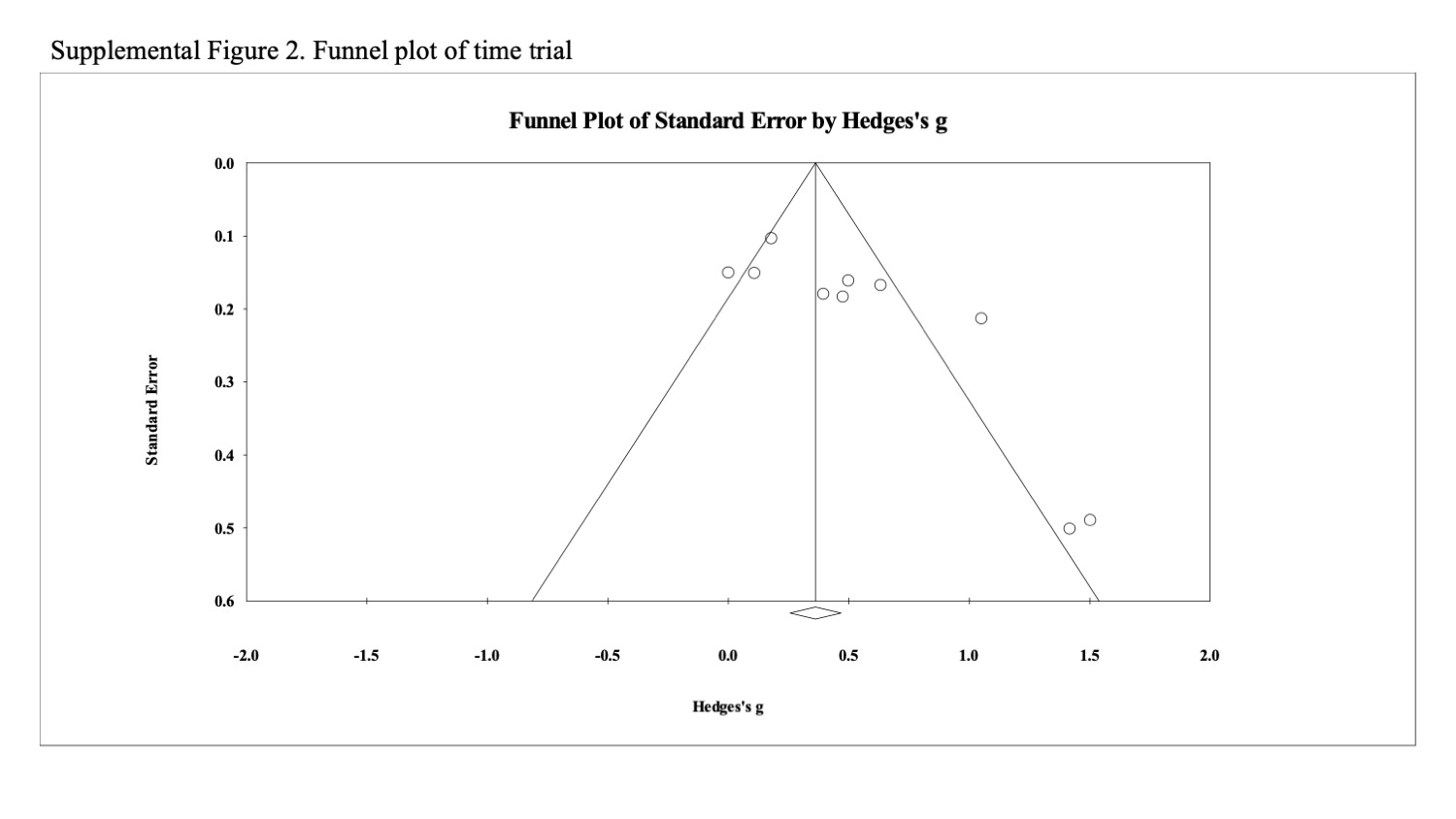

Supplement: Supplementary file 2 [file Image_2.TIFF]

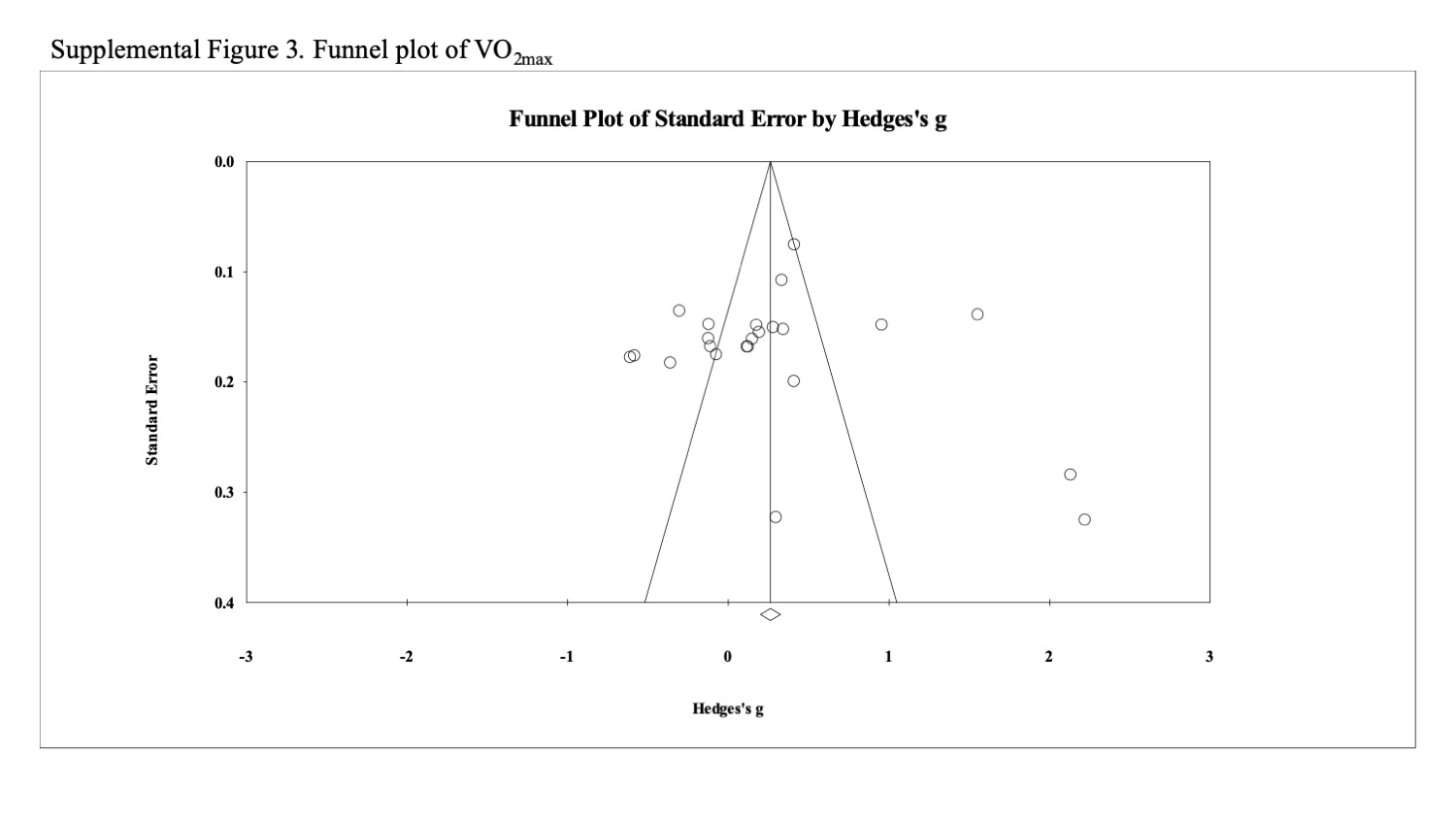

Supplement: Supplementary file 3 [file Image_3.TIFF]

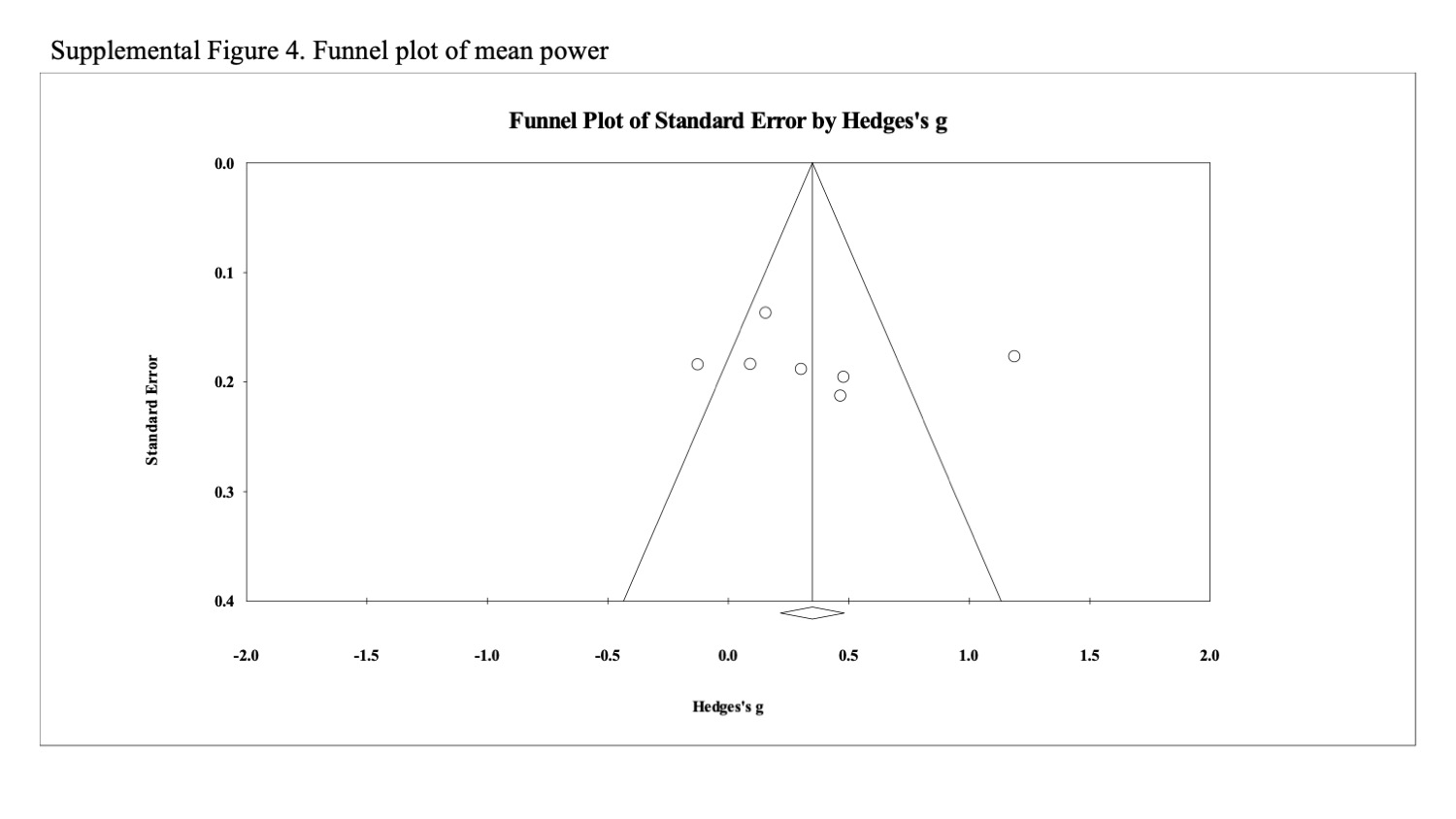

Supplement: Supplementary file 4 [file Image_4.TIFF]

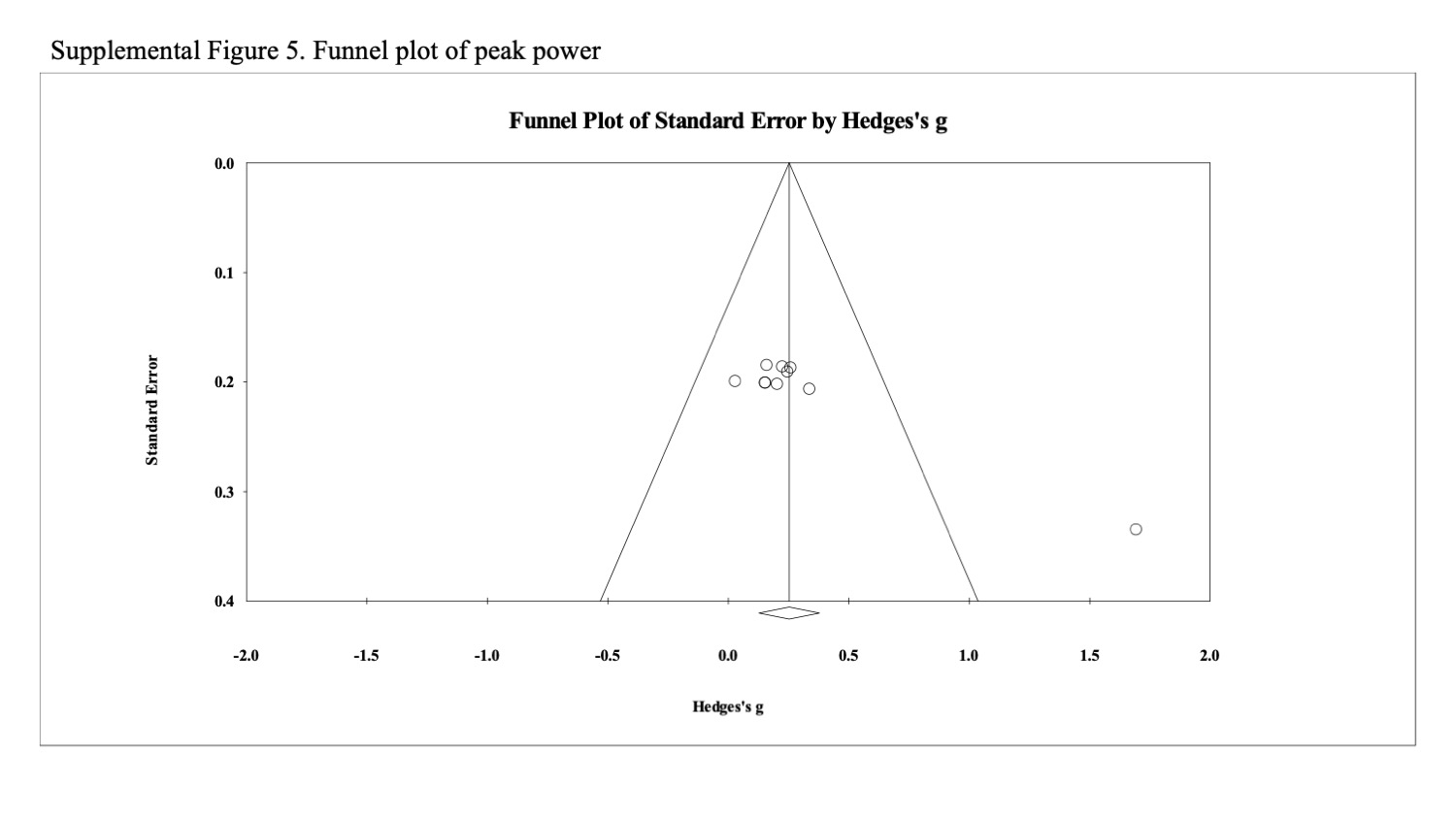

Supplement: Supplementary file 5 [file Image_5.TIFF]
